# A Case of Puerperal Convulsions*In a conversation with a medical friend not long since, on the subject of puerperal convulsions, I had occasion to refer to this case. I was present during the labor and witnessed a number of the convulsions, the termination of the labor and the subsequent recovery of the patient. As it occurred to me that it might prove interesting and instructive to the readers of the Journal, I wrote to my friend, Dr. Field, who has kindly sent me a full report of the case for publication, for which he will please accept my thanks, with those of the other editors. Will not some of my other friends in that excellent Institutions favor us with an occasional article? E. A. A.

**Published:** 1853-12

**Authors:** P. S. Field

**Affiliations:** One of the Resident Physicians in the Baltimore Alms House


### IOWA MEDICAL JOURNAL.

VOL. I. KEOKUK, IOWA, DECEMBER, 1853. NO. 5.

### ORIGINAL COMMUNICATIONS.

### *A case of Puerperal Convulsions.**

BY P. S. FIELD, M. D.,

One of the Resident Physicians in the Baltimore Alms House.

Isabella Sharky, aged 21—native of Ireland—entered the Lying-in Room, pregnant, December the 3d, 1852.

December 9th was taken with labor pains; which continued, with slight intermissions, till ihe morning of the 10th. She complained of severe cramps in the lower extremeties, commencing at the great toe; shortly after which she was seized with convulsions. I did not see her until after the second paroxysm. I then immediately opened a vein in her arm, obtaining, however, but little blood. Ordered cups to the temples and back of the neck, and the following:

℞ Ol. Terebinth, ℥i,

" Ricini, ℥ij,

Muc. G. Acaciae Oj,

M. ft. Enema.

Also rubefacient lotions to the extremeties. Ordered the head to be

*In a conversation with a medical friend not long since, on the subject of puerperal convulsions, I had occasion to refer to this case. I was present during the labor and witnessed a number of the convulsions, the termination of the labor and the subsequent recovery of the patient. As it occurred to me that it might prove interesting and instructive to the readers of the Journal, I wrote to my friend, Dr. Field, who has kindly sent me a full report of the case for publication, for which he will please accept my thanks, with those of the other editors. Will not some of my other friends in that excellent Institutions favor us with an occasional article? E. A. A.

shaved and an evaporating lotion to be kept constantly applied to the same.

On making an examination per vaginam, I found the os tincæ very little dilated, the head presenting. The injection having failed to operate, I ordered another of warm water, with one drop of croton oil.

2 o'clock, P. M.—Made another examination. Found the os fully dilated, the head entering the lower strait. As she was perfectly comatose and the labor was not progressing, with the advice of Dr. Turner, I applied the forceps and delivered the woman of a fine female child. The delivery was followed by a large evacuation of offensive fæces and considerable hæmorrhage, from the uterus. I at once delivered the placenta, which was slightly adherent, when the uterus became firmly contracted. Pulse 130, small and compressible; still comatose. Ordered warm cloths to the vulva.

7 o'clock, P. M.—Was suddenly called, and found the patient in a strong convulsion. There was considerable hemorrhage from the uterus which I found relaxed. I applied cold and warm water alternately over the uterus, and ordered the following:

℞ Vin Ergotæ, ℥i,

Muc. G. Acaciae, ℥ji,

M. ft. Enema.

Soon after which tne uterus contracted firmly, though there was a slight oozing of blood throughout the night. As she had lost so much blood, I found it necessary to stimulate her during the night.

Dec. 11.—The patient had a small passage, this morning. Pulse 130, respiration 28. She has had no return of convulsions since 11 o'clock, last night, but considerable tendency to coma. Still continued the evaporating lotion to the head, and ordered the following:

℞ Hydrarg. proto-Chlor., gr. viij,
Extr. Colocynth. Co., gr. iij.
" Hyoscyami, gr. j.
M. Ft. pil. j.

Which was followed by a large evacuation.

Dec. 12.—Some improvement, this morning. Pulse 110, respiration 20. Complains of severe pain in the head. Ordered a fly blister (3 by 4 in.) to be placed on the back of the neck. Bowels not operated to-day.

Dec. 13.—The patient rested well last night, and is considerably better to-day. Respiration natural, pulse rather quick. From this time she did well, needing only an occasional purgative.

This case is worthy of attention from the number of convulsions, both before and after birth, (there being no less than twenty-one paroxysms,) and the duration of the comatose state. It was evidently a case of the plethoric variety, the patient being a stout Irish girl, a prima-para, and the convulsions, the result of the labor pains,—hence the indication for blood-letting. Another very interesting feature of tho case, was the peculiar twitching sensation felt in the great toe, preceding the cramps which ushered in the first paroxysm. This seems to be analagous to the *epileptic aura* and appears to be in favor of the *identity of puerperal convulsions with epilepsy.*

